# Impact of Wearing Graduated Compression Stockings on Psychological and Physiological Responses during Prolonged Sitting

**DOI:** 10.3390/ijerph15081710

**Published:** 2018-08-10

**Authors:** Masahiro Horiuchi, Chieko Takiguchi, Yoko Kirihara, Yukari Horiuchi

**Affiliations:** 1 Division of Human Environmental Science, Mt. Fuji Research Institute, Yamanashi 403-0005, Japan; takky.guchifoward@mfri.pref.yamanashi.jp (C.T.); handa-y@mfri.pref.yamanashi.jp (Y.K.); 2 Department of Childhood Education, Kyushu Sangyo University, Fukuoka 8138503, Japan; yhoriuti@ip.kyusan-u.ac.jp

**Keywords:** profile of mood states, saliva cortisol, heart rate variability, visual analogue scale, subjective feelings

## Abstract

We investigated the impact of wearing vs. not wearing graduated compression stockings on psychological and physiological responses in 18 healthy young people (12 men and six women) during 3 h prolonged sitting. Profiled of Mood States (POMS) scores did not show marked differences between with and without stockings. A 3 h sit significantly decreased saliva cortisol in both conditions; with no differences between conditions. Wearing stockings suppressed a subjective uncomfortable sensation (e.g., pain; fatigue; swelling) in the lower limbs, as assessed by visual analogue scale (VAS). Increase in heart rate at 1 h and 3 h was significantly greater without than with stockings. In addition, high-frequency oscillations (HF: 0.15–0.4 Hz), used as an indicator of parasympathetic nerve activity, showed higher values with than without stockings throughout the 3 h sitting period—significantly higher at 1 h. When data for both conditions were pooled pre-to-post changes in saliva cortisol were positively associated with higher uncomfortable sensations of VAS in the lower limbs and negatively associated with changes in the Vigor subscale of POMS. Collectively, these findings suggest that wearing graduated compression stockings may benefit from subjective comfort and increased parasympathetic nerve activity.

## 1. Introduction

It has been reported that people with sedentary jobs sit more and stand less on work days than on leisure days [1]. Prolonged sitting time is associated with increased risk of all-cause and cardiovascular diseases mortality, regardless of physical activity level [2,3]. In this regard, several studies have investigated ways to reduce risk of cardiovascular diseases through interventions during prolonged sitting. For example, interrupting prolonged sitting with a few minutes of activity breaks [4,5,6] or with standing [7] significantly reduced postprandial glucose responses when compared with uninterrupted sitting. Similarly, as compared with 7 h uninterrupted sitting, light-intensity walking interruptions suppress elevations in postprandial lipids [8,9]. In addition, regularly breaking up during 7 h prolonged witting with walking of light or moderate intensity reduced systolic and diastolic blood pressure in overweight/obese adults [10].

Aside from the metabolic benefits, physical activity may also have psychological benefits. A previous study found that intermittent light-walking breaks resulted in an attenuation of fatigue levels, as assessed by a visual analogue scale (VAS) during uninterrupted sitting [11]. Additionally, it is reported that use of sit-stand workstations can cause more relaxed, calmer, less sluggish, and higher overall sense of well-being when compared to the only sedentary behavior [12], and that active rest during lunch time at workplace improved mental health in Japanese workers [13]. A recent review also found that an intervention of physical activity can improve psychological well-being in employees [14]. However, it appears that studies of benefits of physical activity to break up sitting have focused on metabolism, and that evidence on psychological responses during prolonged witting is limited. For example, the current review with regard to a relationship between psychological well-being and physical activity conducted for 1326 participants [14], while those with regard to a relationship between sitting time and a risk of metabolic disease, including obesity conducted for more than 50,000 participants [15,16]. Nevertheless, links between sedentary behavior (prolonged sitting or television viewing, computer working in the office) and poor mental health have been found [17,18].

A usage of compression garment may have some potential psychological benefits during prolonged sitting. For patients with lymphedema, wearing compression stockings supported a better quality of life [19], attenuated perceived muscle soreness [20], and rate of perceived exertion in trained populations [21]. It also reduced pain and swelling in deep vein thrombosis (DVT) patients [22]. Since calf circumference, which is a surrogate of swelling and/or venous pooling, was increased during prolonged sitting [23], there is a possibility that wearing compression stockings may benefit mental status and reduce uncomfortable feeling (e.g., pain) in lower limbs during prolonged sitting. Regarding to a prolonged sitting time at workplace, an epidemiologic study reported that Japanese young adults have the longest sitting time out of 20 different developed countries [24]. This may be due to the longer working times among office workers. Therefore, in addition to a decrease sitting time at workplace, effective countermeasures to reduce psychological stress during sitting at the workplace may be a useful health promotion strategy in Japan. As mentioned above, the lower cost and portability of compression garment can make the methodology more readily available for office workers.

Accordingly, in the present study, we hypothesized that wearing compression garments would improve mental status, reduce pain in lower limbs, and improve psychological markers during prolonged sitting. To test this hypothesis, we used medical graduated compression stocking, which have also been used in some previous studies [25,26,27,28,29]. As evaluations, we measured Profile of Mood States (POMS), heart rate variability (HRV), and saliva stress-related markers.

## 2. Materials and Methods

### 2.1. Participants

All of the procedures that were used in the present study were approved by the ethical committee of the Mt. Fuji Research Institute and were performed in accord with the guidelines of the Declaration of Helsinki. Eighteen healthy young adults—12 men and six women—were recruited from the Health Science University. The subjects’ mean age was 21 ± 1 years [mean ± standard deviation (SD)], height was 169 ± 10 cm, and body weight was 64 ± 10 kg. None had any history of cardiovascular disease or mental illness, and none had been taking any medications that could affect physiological or psychological responses related to stress markers or mood states. After receiving a detailed description and explanation of the study procedures and the possible risks and benefits of participation, each subject signed an informed consent form. They were asked to abstain from consuming caffeinated beverages for 12 h and to not engage in strenuous exercise or consume alcohol for a minimum of 24 h before the experiment.

### 2.2. Study Procedures

**Figure 1:**
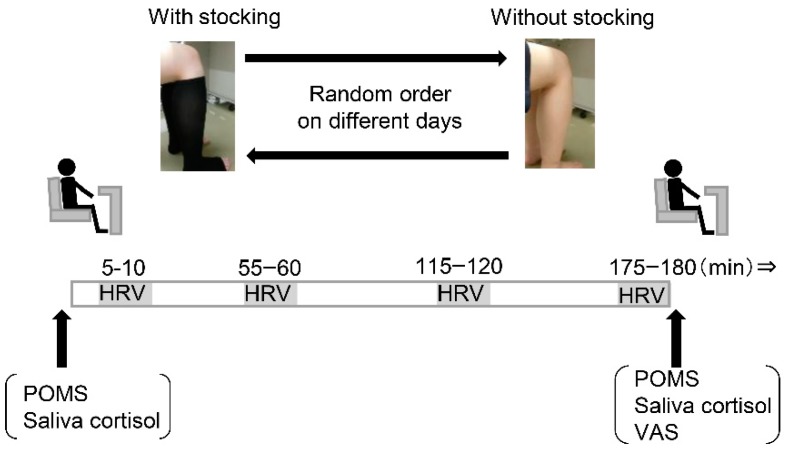
Illustration of experimental procedure. HRV, heart rate variability; POMS, Profile of Mood States; VAS, visual analogue scale.

After 30 min resting period beginning upon arrival to the laboratory, in a supine position on a bed, participants were instrumented with standard lead II electrocardiogram to assess heart rate (HR) (Daily Care BioMedical, Chungli, Taiwan). Then, for the pre-sit measurements, the participants completed the POMS questionnaire, and their saliva was then collected for further analysis. Next, subjects were positioned in a comfortable chair for sit measurement, with or without graduated compression stockings, and HR was continuously measured for 5 min at around 10 min, 1 h, 2 h, and 3 h into the sitting period. The length of the stocking was from the top of the instep to the calf below the knee. The applied pressure of the graduated compression stockings was 29 hPa at the ankle and 20 hPa at the calf. Several widths of stockings were prepared and an appropriate garment was selected for each participant. Throughout the sitting protocol, participants’ feet were gently supported to avoid passive muscle contraction. Study personnel monitored participants during this time to ensure that no leg movement occurred, but participants were allowed to use their arms to read or use a laptop between HR measurements. After 3 h sitting, they completed the POMS questionnaire, their saliva was collected, and they completed the VAS scale for the assessment of uncomfortable feelings, including pain, fatigue, self-perceived sensation of swelling of the lower limbs, or whole body. The study protocol is shown in Figure 1.

### 2.3. Measruements

HR was measured using a Check-My-Heart handheld HRV device (Daily Care BioMedical) [30,31]. The electrodes of the device were attached to the subject’s lower left rib and right clavicle while using a lead electrocardiogram (ECG) signal. The recordings were transferred to a computer, and the data for each 5 min ECG signal were analyzed automatically by HRV analysis software (Daily Care BioMedical). The 5-min HRV data were used in the statistical analysis at each period (i.e., 5–10, 55–60, 115–120, and 175–180 min into the 3 h sitting period). In the frequency domain, the extent of very-low-frequency oscillations (VLF: 0.0033–0.04 Hz), low-frequency oscillations (LF: 0.04–0.15 Hz), and high-frequency oscillations (HF: 0.15–0.4 Hz) was quantified using the fast Fourier transformation [32]. Thereafter, HF power was defined as an indicator of parasympathetic nerve activity, and the ratio of LF to HF values was used as an indicator of sympathetic nerve activity. Saliva for the analysis of cortisol was collected using a Salivette device (No. 51.1534; Sarstedt AG & Co. KG, Numbrecht, Germany), for a 5-min period before and after 3 h sitting. After collection, saliva samples were frozen and subsequently analyzed by SRL Co. Ltd. (Tokyo, Japan). The POMS is a well-established, factor-based, and analytically derived measure of psychological distress. Its reliability and validity have been well documented [33]. The POMS measures six mood states: Tension-Anxiety (T–A), Depression-Dejection (D), Anger-Hostility (A–H), Vigor (V), Fatigue (F), and Confusion (C). We used raw scores from the short Japanese version of the POMS with 30 items [34] for our statistical analysis. The VAS assessed subjective uncomfortable feelings in the lower limbs and whole body. It consisted of a straight horizontal line of a fixed length of 100 mm. The ends were defined as the extreme limits of the parameter to be measured, oriented from the left (best) to the right (worst). In the present study, we set left as no uncomfortable feeling, including pain, fatigue, swelling, and right as intolerable uncomfortable feeling, including pain, fatigue, and swelling. The subject marked the point on the line that they felt best represented their state during 3 h sitting. The VAS score was determined by measuring in millimeters from the left-hand end of the line to the point that the subject marked.

### 2.4. Data Analysis and Statistics

The HRV values for the first 5 min and the last 5 min of each condition were compared. The data are expressed as mean value ± SDs. All statistical analyses were performed using R ver. 2.13.1 (R Core Team, Vienna, Austria). A two-way (Condition × Time) repeated-measures analysis of variance (ANOVA) was used for comparisons of POMS, saliva cortisol, and HRV, and a Bonferroni post-hoc test was employed. A paired t-test was conducted for comparisons of VAS. To estimate the relationship among VAS, changes in saliva cortisol and in subscales of POMS (difference between pre- and post- 3 h sitting), Pearson correlation coefficients were conducted. A *p* value less than 0.05 was considered to be statistically significant. 

## 3. Results

Table 1 shows the changes in the subscale scores of the POMS across with or without stockings, and the summarized results of two-way repeated measures ANOVA are shown in Table 2. Repeated-measures ANOVA revealed a significant main effect of condition (with vs. without stockings) only on the V subscale, and a significant main effect of time (pre vs. post) on the TA, D, V, and F subscales. According to Bonferroini post-hoc test, subscale scores for TA with stocking, and V without stockings significantly decreased post-sitting. 

**Figure 2:**
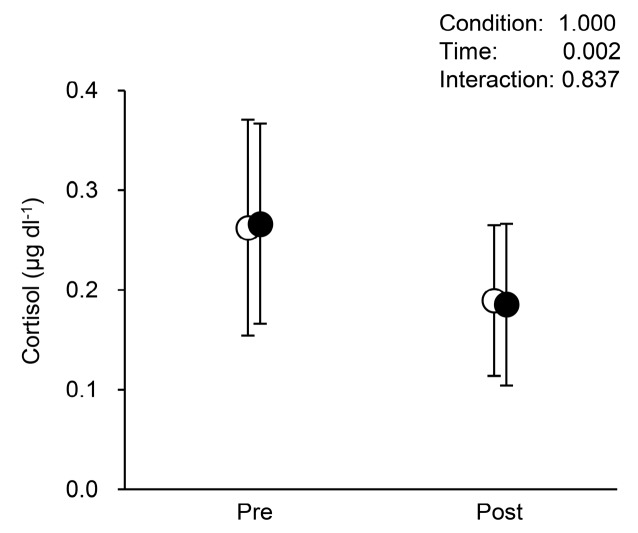
Changes in saliva cortisol, with vs. without stockings. Values are mean ± standard deviation (SD). White circles indicate without stockings and black circles indicate with stockings.

Changes in saliva cortisol across conditions are shown in Figure 2. A significant main effect of the time was observed (F (1, 17) = 13.348, *p* = 0.002, effect size = 0.886, partial *η*^2^ = 0.440, 1 − *β* = 0.931), while no significant main effect of condition was observed (F (1, 17) = 0.000, *p* = 1.000, effect size = 0.000, partial *η*^2^ = 0.000, 1 − *β* = 0.050). There was no significant interaction (F (1, 17) = 0.044, *p* = 0.837, effect size = 0.051, partial *η* ^2^ = 0.003, 1 − *β* = 0.056). A 3 h sit significantly decreased saliva cortisol in both conditions, and the values were very similar.

**Figure 3:**
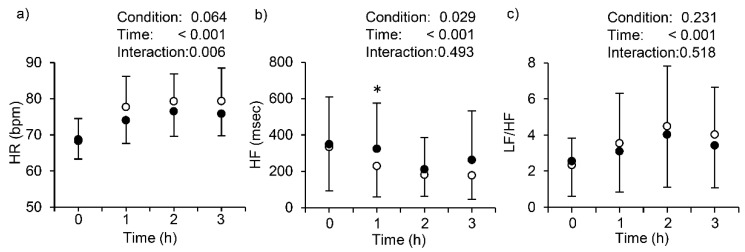
Changes in heart rate (HR; panel **a**) and HR variability (HF; panel **b** and LF/HF; panel **c**) with vs. without stockings. Values are mean ± SD. Each symbol means the same as in Figure 2. HR, heart rate; bpm, beats per minute; HF, high-frequency oscillations, LF/HF, the ratio of low- and high-frequency oscillations. The 0 on the X axis indicates the point between 5 and 10 min into a 3-h sit. * indicates a significant difference between with and without stockings at 1 h.

Figure 3 shows the changes in HR, HF, and LF/HF across with or without stockings, and the summarized results of two-way repeated-measures ANOVA are shown in Table 3. Repeated-measures ANOVA revealed a significant main effect of time in all variables, a significant main effect of condition in HF, and a significant interaction between time and condition in HR. HR increased until 2 h, and it remained stable at 3 h. Wearing compression stockings suppressed an increase in HR when compared to without stockings. HF decreased until 2 h, and values at 1 h with stockings were significantly higher than without, by Bonferroni post-hoc test. By contrast, LF/HF in both conditions increased up to 2 h, and then slightly decreased, but there was no statistical differences between conditions. 

**Figure 4:**
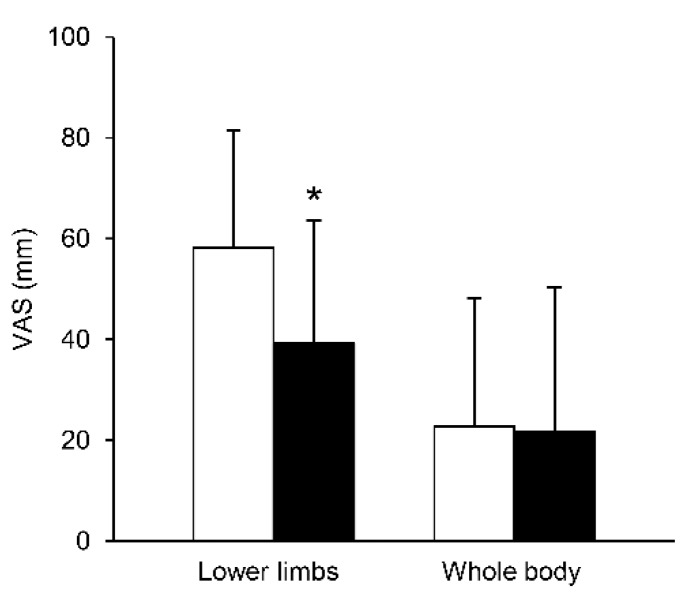
Comparisons in lower limbs and whole body VAS, with vs. without stockings. Values are mean ± SD. White bars indicate without stockings and black bars with stockings. VAS, visual analogue scale. * indicates significant difference between conditions in lower limbs VAS.

Figure 4 compares the lower limbs and whole-body VAS, with vs. without stockings. There was a significant difference in lower limbs VAS between the conditions (*t* = 4.26, *df* = 17, *p* < 0.001, effect size = 1.00, 1 − *β* = 0.98), while no statistical difference was observed in whole body VAS between the conditions (*t* = 0.14, *df* = 17, *p* = 0.894, effect size = 0.03, 1 − *β* = 0.05).

**Figure 5:**
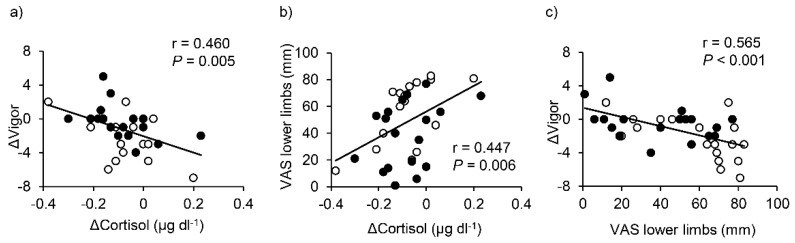
Relationships between changes (Δ) in saliva cortisol (difference between pre–and post–3 h sitting) and changes in Vigor subscale of Profile of Mood Scale (panel **a**), between changes in saliva cortisol and lower limbs VAS (panel **b**), and between lower limbs VAS and changes in Vigor (panel (**c**). Data for both conditions are pooled; thus, sample size was 36 as 18 participants × two conditions. Each symbol means the same as in Figure 3.

Pre-post changes in saliva cortisol were associated with changes in the V subscale of the POMS and lower limbs VAS when the data for both conditions were pooled (Figure 5, panels (a) and (b)). Additionally, changes in V were associated with lower limbs VAS (Figure 5c).

## 4. Discussion

The major findings of the present study are that wearing compression stockings may have more positive effects on subjective feeling of comfort, especially in the lower limbs, and HR responses, as indirect indices of autonomic nervous system. Moreover, pre-post changes in saliva cortisol, as a marker of psychological stress, were associated with lower limbs VAS and changes in the Vigor subscale of POMS, independent of wearing or not wearing compression stockings. These results were partly supported with previous studies in women, showing that an appropriate clothing pressure modulated autonomic nerve system, including HR [35,36], and that wearing compression legwear did not induce any stress during 4-h prolonged sitting [37].

We found significant decreases in TA scores on POMS with stockings, while they were unchanged without stockings. A previous study has demonstrated that sedentary behavior (i.e., prolonged sitting, such as that characteristics of viewing television or working at the computer) is associated with poor mental health, in particular with anxiety [38]. Thus, our results may indicate that acute wearing compression stockings can reduce anxiety that is induced by prolonged sitting. One possible explanation may relate to the autonomic nervous system. Several studies have demonstrated that TA on POMS and sympathetic nerve activity, as assessed by HRV change in parallel [31,39,40,41], although in different experimental settings from the present study. We found higher HF with than without stockings throughout the 3-h sitting period, with significant difference at 1 h. We also found that wearing stockings suppressed increases in HR. These results indicates that parasympathetic nerve activity may be enhanced with stockings. Although the causal relationship between changes in TA and sympathetic nerve activity is unclear, our results may partly account for those of the above-cited previous studies [31,39,40,41]. The subscale scores of V on the POMS significantly decreased without stockings, while those with stockings were well maintained even after 3 h sitting. However, since the pre-values of V without stockings were significantly higher than with stockings, caution is needed to interpret the different results for V between the conditions.

The 3-h sit significantly decreased saliva cortisol levels, without any differences between the conditions. It is well known that cortisol is released by the hypothalamic–pituitary–adrenal axis in response to stress [42], and it is a reliable indicator of endocrine stress responses [43]. Although it is uncertain why no differences in saliva cortisol were found between conditions here, a recent study found that doing deskwork while sitting throughout the slightly decreased saliva cortisol in comparison to doing nothing while sitting [44]. This may indicate that sitting per se cannot reduce psychological stress. Despite this, our results, therefore, may have two possibilities; (1) wearing stockings may not be effective to reduce saliva cortisol, and (2) the 3-h sitting in the present study may not elicit sufficient stress.

In the present study, wearing compression stockings significantly reduced subjective lower limbs discomfort, as assessed by VAS. A recent study demonstrated that compression garments did not produce any beneficial effects on subjective scores from the acute recovery and stress scale [45]. Thus, our results may not be consistent with the previous study [45]; however, the study settings between our and their study are completely different (i.e., after sprint exercise in their study and prolonged sitting in the present study), making it very difficult to interpret these discrepancies. It has been reported that wearing stockings reduces pain and/or swelling in DVT patients, and it is likely that this manipulation may have also reduced swelling here, resulting in lower VAS scores. Although we did not measure volume of lower limbs as an indicator of swelling, a recent study found that 3 h prolonged sitting significantly increased calf circumference, a surrogate of venous pooling [23]. Therefore, it seems that the condition without wearing compression stockings enhanced swelling in comparison to wearing them, while a compression stockings with graduated pressure might facilitate venous return, resulting in lower leg VAS scores. 

In addition to HR responses, other physiological responses should be considered as well as the enhancement of parasympathetic nerve activity. During prolonged sitting, mean arterial pressure and HR have been shown to slightly but significantly increase [23]. As blood pressure is calculated by multiplying the stroke volume by HR via total vascular resistance, the increased HR observed without stockings in the present study may be related to compensation for decreases in stroke volume, and/or vascular resistance to maintain blood pressure. Unfortunately, we cannot presently clarify these interrelationships, as we did not measure these variables; however, it is known that postural change from supine to standing significantly decreases cardiac output [46], and that muscle tensing during standing slightly increases stroke volume and cardiac output and decreases HR, without changes in total vascular resistance. It has been suggested that these integrated physiological responses may have an important role in maintaining BP during standing [46]. Moreover, wearing compression stockings has been suggested to increase venous return during exercise, with increased stroke volume and concomitant reduction in HR [47]. In the present study, although the subjects did not exercise but remained sitting for 3 h, it may be still possible that wearing stockings increased venous return from limbs to heart, resulting in the maintenance of stroke volume and suppression of HR increases. 

Particularly interesting findings of the present study are that changes in saliva cortisol were negatively related with changes in V and positively related with lower limbs VAS. Moreover, higher lower limbs VAS caused greater decreases in V after 3 h sitting. These results suggest that a greater increase in saliva cortisol after 3 h sitting may relate to impairment of subjective vigor and an increase in uncomfortable feeling at lower limbs. Our findings may thus involve an important issue: the relationship between psychological stress and subjective feelings of vigor and lower limbs VAS, irrespective of wearing compression stockings. In this light, links between elevated psychological stress and increased risk of atherosclerosis [48] and acute cardiovascular events [49] have been suggested; however, the mechanisms underlying these links are complex and future studies are required.

We must acknowledge a potential limitation: including both men and women in the present study. It has been reported that menstrual cycle affects saliva cortisol responses [50]; thus, a previous study measured endocrine parameters for women in the early follicular phase of the cycle, which can minimize the effects of sex hormone [51]. Moreover, leg venous compliance as a determinant of peripheral venous pooling differs markedly between men and women, probably due to different sympathetic outflow [52]. However, measures were taken to mitigate this limitation: the women in the present study had regular menstrual cycles and were not taking hormonal contraceptives; moreover, to minimize sex hormone effects, based on the previous study [51], all measurements of women were performed during days 1–5 of their menstrual cycle. 

## 5. Conclusions

In summary, our results suggest that 3 h prolonged sitting reduced psychological stress, as evaluated by saliva cortisol, irrespective of wearing or not wearing compression stockings. However, wearing compression stockings may have beneficial effects for a subset of subjective feelings of TA on POMS, and lower limbs VAS and for autonomic nerves system by enhancing parasympathetic nerve activity. Moreover, the magnitude of increases in saliva cortisol was associated with the magnitude of decreases in V and increases in lower limbs VAS when the data of both conditions were pooled together. Collectively, the present results may therefore provide helpful information and guidance for future research into the benefits of wearing compression stockings during prolonged sitting.

## Figures and Tables

**Table 1:** Changes in Profile of Mood States scores across with or without compression stockings.

	Without Stockings		With Stockings		*p*-Value		
	Pre	Post	Pre	Post	Condition	Time	Interaction
TA	2.1 ± 2.9	1.7 ± 2.5	1.6 ± 2.5	0.7 ± 1.3 ^†^	0.146	0.035	0.276
D	0.8 ± 1.2	0.4 ± 0.8	0.8 ± 1.5	0.4 ± 0.7	0.875	0.038	0.868
AH	0.7 ± 1.6	0.2 ± 0.5	0.2 ± 0.5	0.2 ± 0.5	0.104	0.154	0.298
V	4.8 ± 3.3	2.6 ± 3.1 ^†^	2.9 ± 2.7 *	2.5 ± 3.5	0.019	0.013	0.006
F	1.1 ± 1.7	2.7 ± 3.2	0.9 ± 2.0	1.7 ± 1.9 *	0.060	0.025	0.127
C	4.4 ± 1.5	3.9 ± 1.2	4.2 ± 1.4	4.1 ± 1.2	0.901	0.077	0.269

Values are mean ± standard deviation (SD). TA, tension-anxiety; D, depression-dejection; AH, anger-hostility; V, vigor; F, fatigue; C, confusion. * *p* < 0.05 between with or without stockings within the same time period. ^†^ *p* < 0.05 between pre and post within the same conditions.

**Table 2:** Summarized results of statistical analysis for profile of mood states.

	Main Effect								Interaction			
	Condition				Time				Condition × Time			
	(With or without Stockings)				(Pre vs. Post)							
	F	ES	P*η*^2^	1 − *β*	F	ES	P*η*^2^	1 − *β*	F	ES	P*η*^2^	1 − *β*
TA	2.33	0.37	0.12	0.30	5.23	0.56	0.24	0.58	1.28	0.27	0.07	0.19
D	0.03	0.04	0.00	0.05	5.05	0.55	0.23	0.56	0.03	0.04	0.00	0.05
AH	2.96	0.42	0.15	0.37	2.23	0.36	0.12	0.29	1.15	0.26	0.06	0.17
V	6.77	0.63	0.29	0.69	7.78	0.68	0.31	0.75	10.03	0.77	0.37	0.85
F	4.06	0.49	0.19	0.48	6.07	0.60	0.26	0.64	2.58	0.39	0.13	0.33
C	0.02	0.03	0.00	0.05	3.54	0.46	0.17	0.43	1.31	0.28	0.07	0.19

Degrees of freedom for the main effect of condition, time, and interaction are (1, 17). ES, effect size; P*η*^2^, partial *η*^2^; 1 − *β*, statistical power.

**Table 3:** Summarized results of statistical analysis for heart rate variability.

	Main Effect								Interaction			
	Condition				Time							
	(With or without Stockings)				(Pre vs. Post)							
	F	ES	P*η*^2^	1 − *β*	F	ES	P*η*^2^	1 − *β*	F	ES	P*η*^2^	1 − *β*
HR	3.92	0.48	0.19	0.46	44.64	1.62	0.72	1.00	4.73	0.53	0.22	0.87
HF	5.68	0.58	0.25	0.61	8.56	0.71	0.34	0.99	0.81	0.22	0.05	0.21
LF/HF	1.54	0.30	0.08	0.22	9.09	0.73	0.35	0.99	0.77	0.21	0.04	0.20

Degrees of freedom for the main effect of condition are (1, 17), and for main effect of time and interaction (3, 51). HR, heart rate; HF, high-frequency oscillations; LF/HF, the ratio between low- and high-frequency oscillations.
